# Carbocycle-Based Organogelators: Influence of Chirality and Structural Features on Their Supramolecular Arrangements and Properties

**DOI:** 10.3390/gels7020054

**Published:** 2021-05-01

**Authors:** Rosa M. Ortuño

**Affiliations:** Departament de Química, Universitat Autònoma de Barcelona, 08193 Cerdanyola del Vallès, Spain; rosa.ortuno@uab.es

**Keywords:** low-molecular-weight organogelators, cycloalkanes, cholesterol-based organogelators, chirality, stimuli-responsive materials, self-healing gels

## Abstract

The rational design and engineer of organogel-based smart materials and stimuli-responsive materials with tuned properties requires the control of the non-covalent forces driving the hierarchical self-assembly. Chirality, as well as *cis/trans* relative configuration, also plays a crucial role promoting the morphology and characteristics of the aggregates. Cycloalkane derivatives can provide chiral chemical platforms allowing the incorporation of functional groups and hydrophobic structural units able for a convenient molecular stacking leading to gels. Restriction of the conformational freedom imposed by the ring strain is also a contributing issue that can be modulated by the inclusion of flexible segments. In addition, donor/acceptor moieties can also be incorporated favoring the interactions with light or with charged species. This review offers a perspective on the abilities and properties of carbocycle-based organogelators starting from simple cycloalkane derivatives, which were the key to establish the basis for an effective self-assembling, to sophisticated polycyclic compounds with manifold properties and applications.

## 1. Introduction

Nowadays, the applications of organogels as soft materials are huge in several areas. They include rationally designed gels for use in drug formulation and development [1], drug release [2], medical applications [3], encapsulation and controlled flavor and fragrance release [4], pollutants removal [5], supramolecular electronics [6], materials for bioinspired catalysis [7], selective agents for anion recognition [8,9], materials for eco-friendly oil spill recovery [10,11,12] and products with manifold properties such as tissue regeneration materials, biosensors, cosmetics and lubricants, among other applications [13,14,15].

The design of low-molecular-weight organogelators (LMWOGs) with specific properties and targeted functions has been a real challenge for a highly active research field during the last 20 years and remains far from generalization [16,17,18]. The control of the driving forces involved in self-assembling of organogelator molecules, i.e., hydrogen bonding and other non-covalent interactions such as van der Waals, π–π, dipolar and Coulomb interactions, is crucial to achieve hierarchically ordered architectures. In addition, chirality plays a key role in the formation of aggregates [19,20]. In addition, precise correlation of the non-covalent interactions with bulk properties and corresponding functions allows to engineer smart materials [21] and multi-stimuli responsive materials [22,23,24,25,26].

Carbocyclic rings can act as chemical platforms bearing suitable functional groups, such as amide, urea or amino acid, which are able to form hydrogen bonds, as well as long alkyl chains or aromatic systems favoring molecular stacking. Furthermore, in these molecules, chirality, in conjunction with conformational restriction due to the ring strain, is a powerful tool to promote and direct the aforementioned interactions leading to aggregates with specific characteristics.

In this review, a perspective from highly relevant forerunner works till very recent results that deal with the gelling ability and self-assembling of carbocycle-based organogelators is afforded. The role of the chirality in the monomers and in the aggregates is emphasized. Special attention has been paid to compounds containing simple cycloalkane rings, mainly cyclohexane or cyclobutane derivatives. Cholesterol-based organogelators are also discussed because of their versatility allowing to design and prepare tailor-made organogelators with interesting properties.

## 2. Cyclohexane Derivatives

In this section, the gelling abilities of bisamide and bisurea derivatives are extensively commented because they have been used by several authors to shed light on the role of balanced hydrogen bonding and van der Waals interactions as the driving force to produce gels. Attention is paid to the relationship between the chirality of the monomers and that of the aggregates. The influence of the substituents, placed on the cyclohexane ring and of the side-chain length is also considered.

### 2.1. Dialkyl Bisamide and Bisurea-Based Organogelators

The simplest gelators are derivatives of *trans*-1,2-diaminocyclohexane or *trans*-cyclohexane-1,2-dicarboxylic acid. Both present a *C*_2_ axis of symmetry and are commercial products available in the two enantiomerically pure forms.

**Figure 1:**
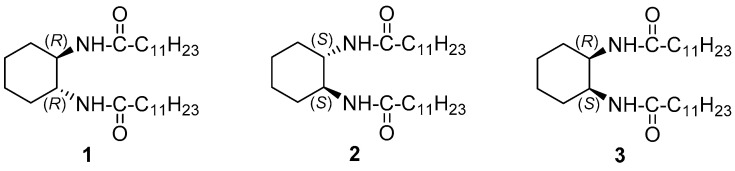
Cyclohexane-containing bisamides based on cyclohexane-1,2-diamine.

In a pioneer work, Hanabusa et al. described the synthesis and study of LMWOGs **1**–**3** (Figure 1) [27]. Compound **1** was prepared by the reaction of (1*R*,2*R*)-1,2-diaminocy- clohexane and lauroyl chloride in the presence of triethylamine. Derivatives **2** and **3** were prepared in a similar way. Compound **1** was able to gelate a variety of solvents of diverse polarity and functionality, such hexane, methanol, ethyl acetate, acetone, acetonitrile, dioxane, toluene and *N,N*-dimethylformamide, affording gels that were stable at 25 °C for six months. A similar behavior was observed for the enantiomer **2**. Interestingly, the racemate only formed unstable gels that were converted to cocrystals after several hours. In turn, the achiral *cis*-derivative **3**, prepared from *cis*-cyclohexane-1,2-dicarboxylic acid, formed no gels with any of the liquids tested. Gelation was dependent of the length of the hydrophobic side chain in **1** since gels fail to form when the alkyl tail was shortened to four carbons.

**Figure 2:**
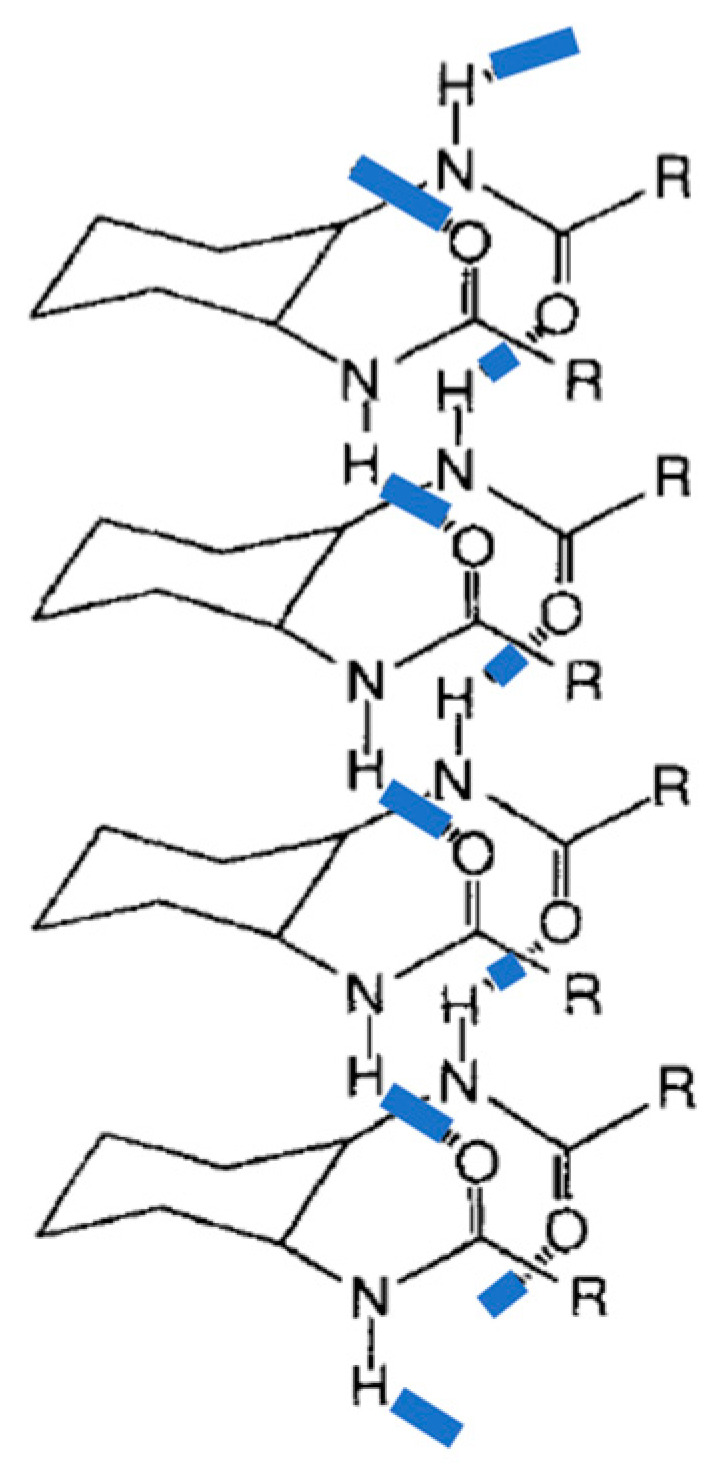
Suggested model to explain the helicoidal aggregates from **1**. Adapted with permission from Wiley [27].

Gels from acetonitrile were investigated by Transmission Electron Microscopy (TEM) revealing nano-sized helical fibers. The helicity was right-handed for (*R,R*)-**1** and left-handed for its enantiomer, (*S,S*)-**2**. As a result of Molecular Modeling (MD) studies, authors suggested that such structures are induced by the formation of two intermolecular hydrogen bonds between each molecule. Thus, the two-equatorial amide-NH and amide-CO could direct themselves antiparallel to each other and perpendicular to the cyclohexyl ring promoting the formation of an extended molecular tape (Figure 2). Although hydrogen bonding between amide groups in consecutive molecules has also been proposed by other authors in later studies to explain the self-assembling mode of related systems (see below), such a cyclohexane stacking has not been supported neither by experimental evidence nor by recent computational studies.

**Figure 3:**
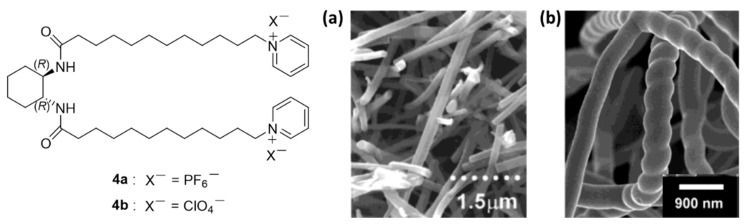
Structure of amphiphilic organogelators **4a** and **4b** and SEM images of (**a**) self-assembled **3a** in ethanol; (**b**) tantalum oxide right-handed fibers obtained from **4b**. Adapted with permission from ACS [28,29].

Later, amphiphilic cationic compounds **4** and their enantiomers were investigated. They formed self-assembled rods in ethanol as verified by Scanning Electron Microscopy (SEM) and TEM (Figure 3a) [28].

These super-structures were used as templates for the preparation of tubular helical fiber materials of transition metal (Ti, Ta, V) oxides. The helices of metal oxides were always left-handed in the sol-gel system of the (*R,R*)-enantiomer, **4b** (Figure 3b) and right-handed in the case of the (*S*,*S)*-enantiomer [29].

**Figure 4:**
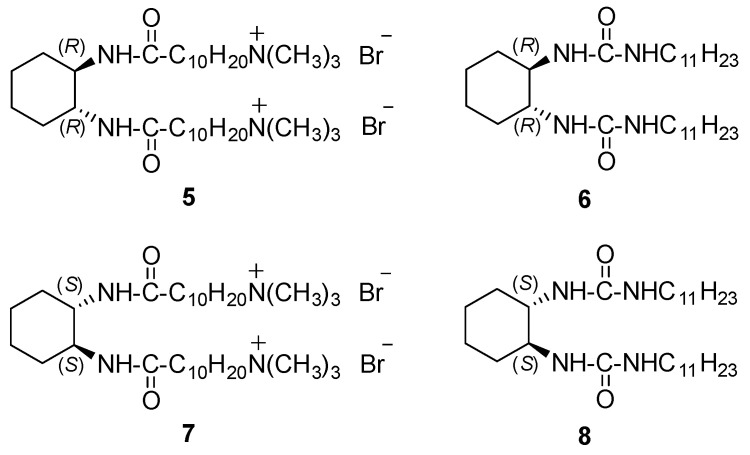
Enantiomeric cationic gelators **5** and **7** and urea-based neutral gelators **6** and **8**.

The preparation of helically structured silica by a sol-gel transcription in chiral organogel systems including bisamide and bisurea derivatives was reported by Shinkai et al. [30,31]. Figure 4 shows the (*R,R*)-enantiomers of cationic amphiphile **5** and bisurea **6**. Bisamides **1** and **2** (Figure 1) were also used as gelators in that work.

Cationic gelators **5** and **7** were worse than related bisamides and bisureas since they were soluble in protic solvents like alcohols and acetic acid or strongly polar aprotic dimethylsulfoxide (DMSO). They gelated aprotic acetone, acetonitrile, tetrahydrofuran (THF), DMF or cyclohexane but were insoluble in n-hexane. In turn, bisureas **6** and **8** behaved similarly to bisamides **1** and **2**. However, they were insoluble in acetone and acetonitrile.

**Figure 5:**
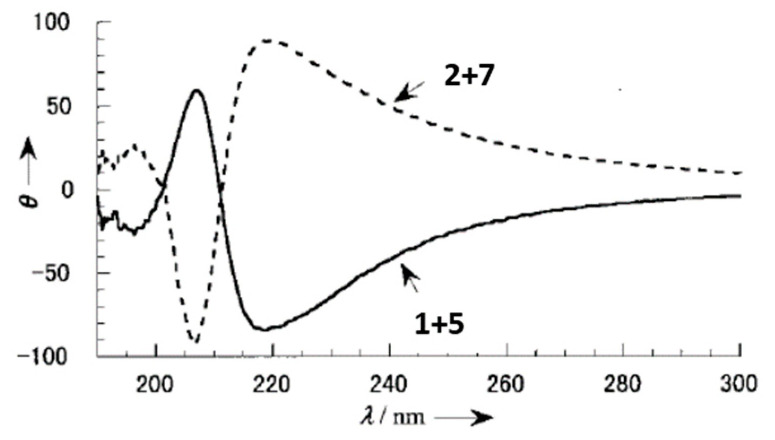
CD spectra of **1** + **5** (1:1 wt %) and **2** + **7** (1:1 wt %) organogels obtained from acetonitrile: 25 °C, total gelator concentration: 4.18 × 10^−3^ mol L^−1^. Adapted with permission from Wiley [31].

Since the presence of the cationic charge in the organogel fibers was as an indispensable feature to efficient sol-gel transcription, mixtures of **1** + **5**, **5** + **6**, **2** + **7** and **7** + **8** were applied as gelators in order to maintain the high gelation ability and moderate cationic charge density. SEM pictures of xerogels of these mixtures (1:1 wt %) from acetonitrile showed nanosized helical fibers for the aggregates. The helicity was left-handed for the mixture **1** + **5** (*R* enantiomer) and always right-handed for **2** + **7** (*S* enantiomer). Contrariwise, for xerogel samples of **2** + **5** (1:1 wt %) obtained from ethanol, helicity was right-handed for the *R* enantiomers and left-handed for the *S* enantiomers. These results were in agreement with the sign of the Cotton effect observed in the Circular Dichroism (CD) spectra of these xerogels (Figure 5).

The transcription of the chiral, helical structures of the organogels into silica gel was achieved by sol-gel polycondensation of tetraethoxysilane (TEOS). In all cases, the left- and right-handed structures of the silica could be created by transcription of the left- or right-handed structures of the organogel systems.

**Figure 6:**
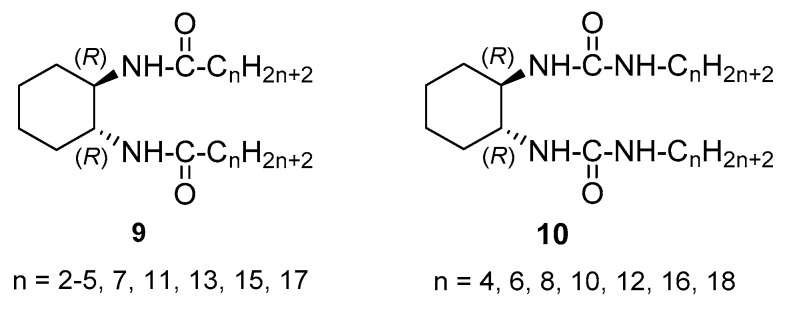
Cyclohexane-based bisamide and bisurea organogelators bearing alkyl chains of different lengths.

Some years later, van Esch et al., investigated on the balanced influence of hydrogen bonding and van der Waals interactions on the gelation of cyclohexane-based bisamide and bisurea derivatives [32]. They prepared compounds **9** and **10** bearing alkyl tails of different lengths (Figure 6) and their gelation behavior with different solvents was explored.

For the bisamide with a *C*_2_-alkyl tail, the formation of intermolecular hydrogen bonds and interactions of the cyclohexyl rings were expected to be the major driving force for gelation, because the van der Waals interactions cannot contribute in a significant manner. Indeed, gelation was not observed in protic solvents capable to interfere with hydrogen bonding but it gelated apolar solvents and was soluble in alcohols and DMSO. In contrast, compounds with *C*_5_–*C*_13_ alkyl tails formed gels with either apolar or polar solvents that can not interfere with hydrogen bonding, i.e., ethyl acetate. With *C*_15_– and *C*_17_– bisamides, gelation also occurred in protic polar solvents, such as isopropanol, in addition, to all the polar and apolar solvents tested. Their critical gelation concentration (*cgc*) was low enough (<0.5 wt %) to classify them as supergelators. In conclusion, the elongation of the alkyl tail in bisamide organogelators led to lower *cgc*, mainly in polar solvents, whereas this effect was less pronounced in apolar solvents.

Bisurea-based gelators were less soluble in apolar solvents than bisamides. Increasing of the tail length allowed the formation of gels either in apolar and polar solvents for *n* = 10–18. *C*_16_–tail bisurea seemed to have the optimal length and showed the lowest *cgc* values being also classified as supergelator.

As a general conclusion of this work, it was demonstrated that for these organogelators, van der Waals interactions play a dominant role in polar solvents whereas hydrogen-bonding interactions dominate in apolar ones. These results point out the significant role of solvent in driving the assembly of the gel fibers.

**Figure 7:**
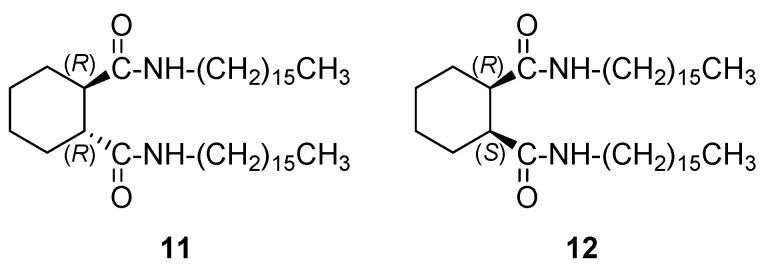
Cyclohexane-containing bisamides based on cyclohexane-1,2-dicarboxylic acid.

The regioisomeric cyclohexane-based bisamides **11** and **12** (Figure 7), that is, derivatives of the cyclohexane-1,2-dicarboxylic acid, related to **1**, **3** and **9**, have also been examined as LMWOGs [33].

These compounds were synthesized by means of standard peptide coupling protocols. Both were insoluble in solvents with high dielectric constants (ε) but chirality played a significant role in the ability to gelate solvents with ε < 6. While achiral meso (*R,S*)-**12** behaved as a moderate gelator, chiral (*R,R*)-**11** was a very good gelator of solvents such as 1,4-dioxane and toluene, with *cgc* = 3 and 7 mg mL^−1^, respectively. These values are like those described by Hanabusa [27] and van Esch [32] for **1** (Figure 1) and **9** (Figure 6), respectively. Hence, regiochemistry in *trans*-cyclohexane-based bisamides seem not to be relevant for the gelation ability of these compounds.

All gels were thermoreversible and stable at room temperature for several months. The sol-gel transition temperature (*T*_gel_) was determined by high resolution ^1^H NMR spectroscopy in *d*_8_-toluene. At the same concentration, **11** gelates at higher temperature (*T*_gel_ = 322 K) and the N*H* chemical shift appears at relatively high field (5.9 ppm). In the case of **12**, *T*_gel_ is lower (290 K) and the N*H* appears deshielded at higher chemical shifts (6.4 ppm). These data suggest that the N*H* proton in **11** is less fixed than in **12** and, therefore, the gelation process is related not only to the intermolecular hydrogen bonding but also to an appropriate preordering of the *C*_16_-alkyl chain. These features were supported and explained by computational calculations that suggested that the supramolecular interactions of these compounds to form ordered aggregates is promoted by a cooperative effect of van der Waals interactions and hydrogen bonding, the latter being predominant.

**Figure 8:**
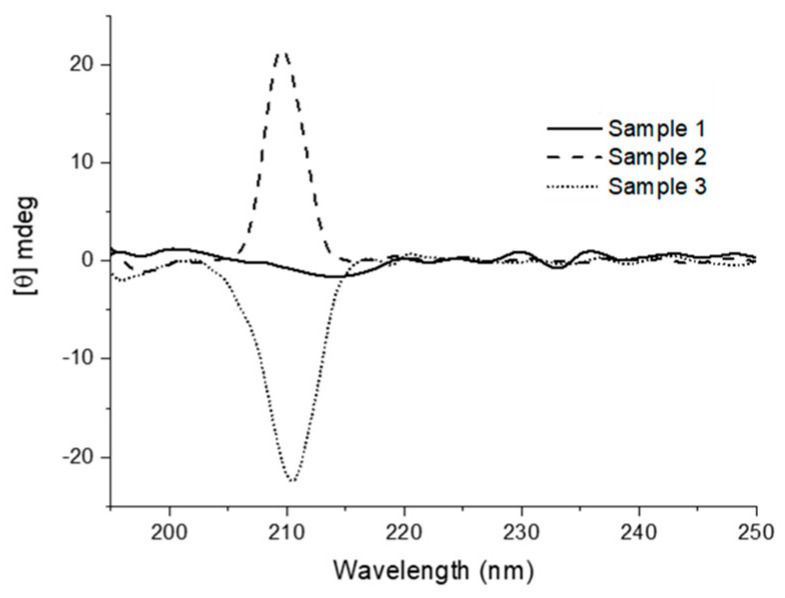
CD spectra of representative samples of xerogels of **12** (from toluene) in KBr at 25 °C. In all cases, the corresponding gels were prepared under sonication. The spectrum for each sample is the average of several measures. Reprinted with permission from Wiley [33].

For the xerogels of both organogelators from toluene, SEM images showed aggregates formed by fibrillar networks. Calculations predicted that, in the two compounds, the amido groups adopt an anti-disposition and that the monomers self-assemble to form supramolecular helixes, right-handed for **12** and left-handed for **11**. This was confirmed by the CD spectra of the corresponding xerogels from toluene, which show a positive band for **11** and was not silent for **12** giving a single band. That means that both aggregates are chiral despite meso compound **12** is achiral. Figure 8 shows that the sense of the chirality is not reproducible seeming that the production of either one enantiomeric assemble or the other is produced at random, giving enantiomerically enriched or racemic aggregates according to a stochastic chiral symmetry breaking [34]. Accounting for the origin of the chirality, was suggested that this case is an example of supramolecular physical asymmetric induction [35] promoted by sonication [36]. Indeed, ultrasound activation was used to promote the fast formation of gels at the *cgc* and, in contrast, CD of xerogels prepared without the help of ultrasounds did not show any chirality. Although there are examples in the literature about the formation of chiral aggregates from achiral monomers in aromatic systems [37] there is not so frequent on the base of simple alkane bisamides as organogelators.

**Figure 9:**
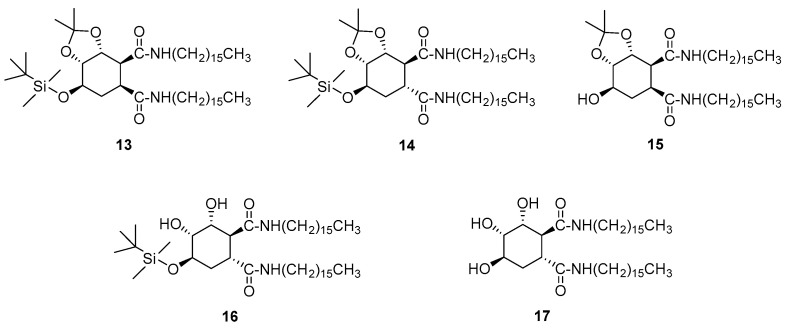
Polyfunctionalized cyclohexane-based bisamide organogelators.

The effect of hydroxyl groups as substituents of the cyclohexane ring on the gelling abilities of cyclohexane-based bisamide organogelators were investigated [38]. *C*_16_-alkyl bisamides derived from trihydroxy cyclohexane-1,2-dicarboxyxlic acid, **13**–**17** (Figure 9), were synthesized from commercially available (–)-shikimic acid [39].

These compounds produced gels in common organic solvents that were stable at room temperature for several weeks. Bisamides **13**–**16** were able to gelate alcohols contrariwise to **11**, **12** and **17** that were insoluble. The influence of *cis/trans* diastereoisomerism for **13** and **14** was less remarkable than it was for **11** and **12**. Compound **15** behaved as a bad organogellator because it was insoluble in nearly all solvents tested, except in 1,4-dioxane and toluene, but it exhibited high *cgc* values (51 and 102 mg mL^−1^, respectively). Functionally, it differs from **13** and **14** in the presence of a free hydroxyl group and this fact clearly disfavors its gelling activity specially in methanol and ethanol. Bisamide **16**, with two free hydroxyl groups, was a better organogellator than **14** and gelated a large variety of solvents of different ε; in addition, it was a good gelator for acetone (*cgc* = 22 mg mL^−1^) while all others, **11**–**15** and **17**, were soluble or insoluble. Derivative **17** behaves like **14** and **15** as organogellator of aprotic solvents, although with significant higher *cgc* values. Nevertheless, despite the presence of three free hydroxyl groups, compound **17** was insoluble in all alcohols tested (methanol, ethanol and isopropanol) and in water.

**Figure 10:**
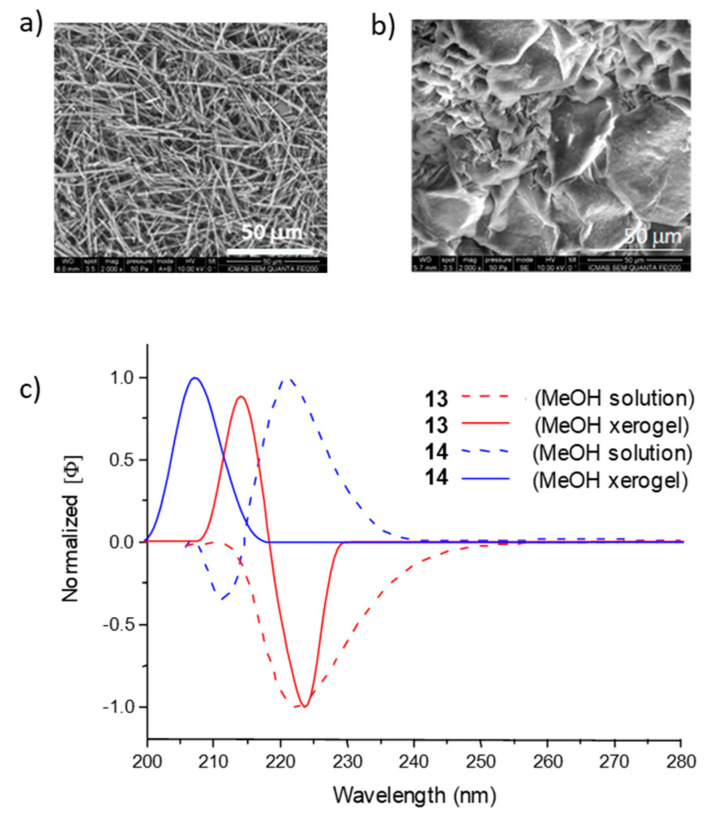
Top: SEM images of xerogels from methanol at the *cgc* of: (**a**) **13**, (**b**) **14**. Magnification 50 µm. (**c**) Normalized CD spectra of **13** and **14**, respectively, in methanol solution (2.4 mM for **13** and 2.7 mM for 14) and as xerogel from methanol in KBr (20 mM) at 25 °C [38].

All these results suggested that the gelling properties for organogelators **13**–**17** depend not only on the dielectric constant of solvents, but, for their understanding, other factors need to be considered. The combined use of Hansen solubility parameters (HSPs) [40,41], SEM, CD and computational calculations afforded a rationale for the main interactions operating in the self-assembling of each organogelator in a determined group of solvents. Thus, for *cis/trans* diastereoisomers **13** and **14**, their SEM images revealed the formation of fibers in the case of **13** and fibrous platelets in the case of **14**, which is in agreement with the arrangements predicted by calculations for each organogelator: curved aggregates without a helicity trend for **13** and right-handed helical aggregates for **14**. CD confirmed these predictions displaying a bisignate spectrum for **13** and a monosignate positive band for **14**, both as xerogels from methanol (Figure 10).

**Figure 11:**
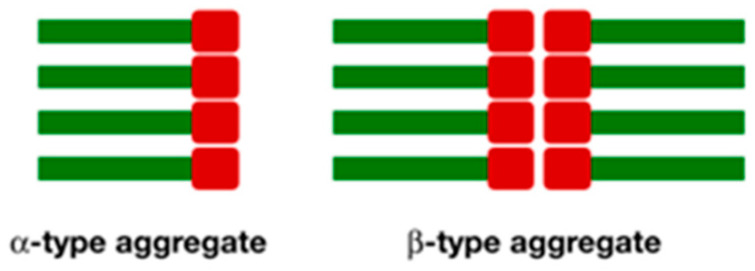
Cartoon representing the spatial disposition of monomers in α- and β-aggregates [38]. Red: polar heads; green: hydrophobic chains.

Considering the other three organogelators, **15**–**17**, compound **15**, owning low hydrogen bonding ability, is a bad organogelator for apolar solvents and it is soluble in polar ones. The CD spectrum of its toluene xerogel agrees with the computed structure that suggests the formation of a right-handed helical aggregate. Organogelator **16**, with two free hydroxyl groups, presents ambivalent activity since two types of aggregation are predicted by calculations depending on the polarity of solvents: α−type, which is described as a vertical aggregation promoted by intermolecular amide hydrogen bonding, in polar solvents; and β−type, where two molecules are facing head-to-head and then piled vertically, in apolar ones (Figure 11). This solvent-mediated ambivalence is corroborated by the different morphologies shown by SEM images of xerogels form acetone (disorganized shapes) and pentane (platelets), respectively, and supported by Hansen parameters and CD.

**Figure 12:**
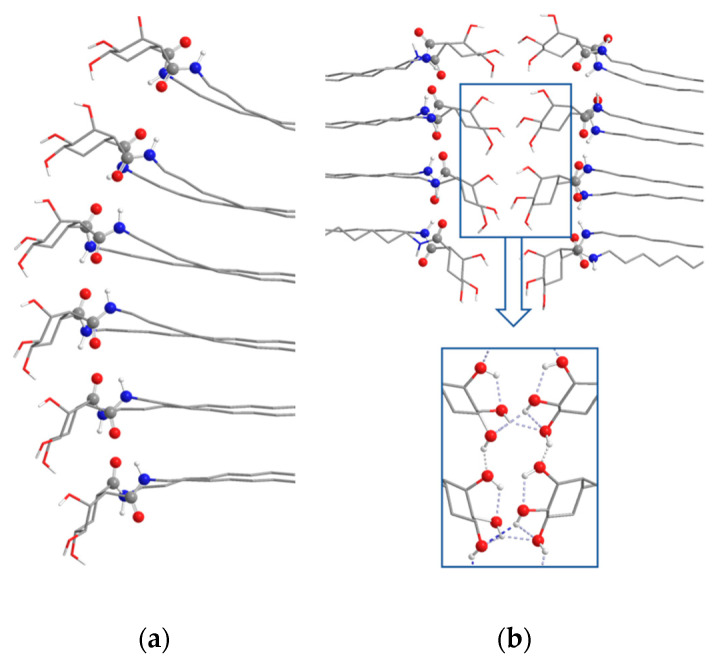
(**a**) Side view of central 6 molecules in octameric 1-D aggregate (amide hydrogen-bonds) **17**-α. (**b**) Side views of octameric 2-D aggregates (amide and hydroxyl hydrogen-bonds) **17**-β. Non-polar hydrogen atoms have been omitted for clarity. Atoms in amide groups have been represented with red (oxygen), blue (nitrogen) and grey (carbon) spheres [38].

Finally, compound **17** with three hydroxyl groups did not interact with very polar solvents due to the formation of intra- and intermolecular hydrogen bonds and, consequently, it is insoluble in alcohols and water; nevertheless, it is able to gelate low polarity solvents by means of no hydrogen-bonding interactions, as modelled by theoretical calculations (Figure 12).

### 2.2. Trisamide-Based Organo- or Hydrogelators

**Figure 13:**
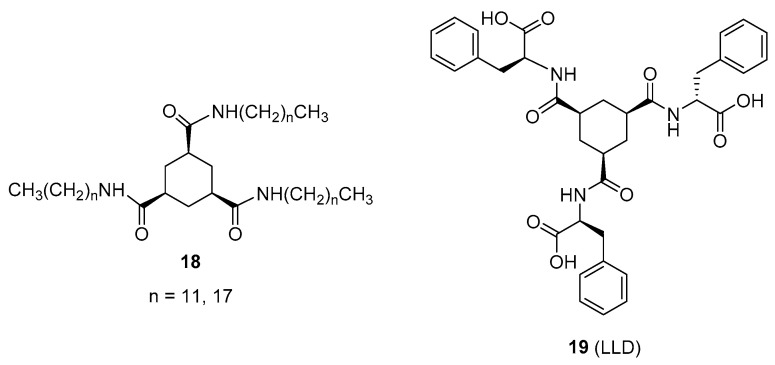
Structures of 1,3,5-cyclohexanetricarboxamide-based organogellators **18** and hydrogelator **19** (LLD).

The ability of trialkyl cis-1,3,5-cyclohexanetricarboxamides **18** (Figure 13) as organogellators was investigated for the first time by Hanabusa et al. [42]. These compounds afforded organogels from apolar solvents, the driving force for gelation being intermolecular hydrogen bonding between amides and van der Waals interaction among hydrophobic alkyl chains.

Later, van Esch et al., explored the effect of the N-substituents in trisamides derived from cyclohexane-1,3,5-tricarboxyxlic acid. The change of the alkyl chains by polar hydrophilic moieties turned the new compounds in good hydrogelators [43]. In particular, derivatives with L- or D-alanine were considered focusing on the influence of the chirality in the amino acid on the gelling properties [44]. As an example, Figure 12 shows compound **19** formed with two L-alanine residues and one D-alanine moiety (LLD). Whereas homochiral derivatives (LLL or DDD) did not gelate water, its heterochiral variants (LLD or DDL) were good hydrogelators. TEM images revealed that LLD and DDL formed nanosized-fiber bundles, which were right-handed for the LLD gel and left-handed for the DDL one. Moreover, peripheral functionalization of the homochiral derivatives LLL or DDD by means of a second amino acid or a hydrophilic moiety overcame the effect of chirality and led to nice hydrogelators.

### 2.3. Bis(Acyl-Semicarbazide)-Based Organogelators

**Figure 14:**
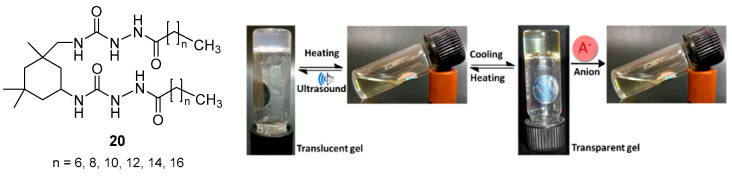
Structure of cyclohexane-based bis(acyl-semicarbazide) gelators **20** and photograph of the multiple switching process of the C_12_-organogellator under the alternate effects of ultrasound, temperature and anions. Adapted with permission from ACS [45].

Cyclohexane-containing bis(acyl-semicarbazide)-based gelators **20** (Figure 14) with an additional amino group incorporated into urea moieties and bearing hydrophobic alkyl chains (*C*_8_–*C*_18_) were prepared by addition of fatty acid hydrazides to a precursor bis(isocyanate) [45]. The gels from aromatic solvents (benzene, toluene, chlorobenzene, *o*-, *m*-, *p*-xylene) were thermoreversible and multi-stimuli-responsive. Indeed, the gels displayed ultrasound-responsive nature and could be tuned in the presence of anions at different concentrations (Figure 12).

**Figure 15:**
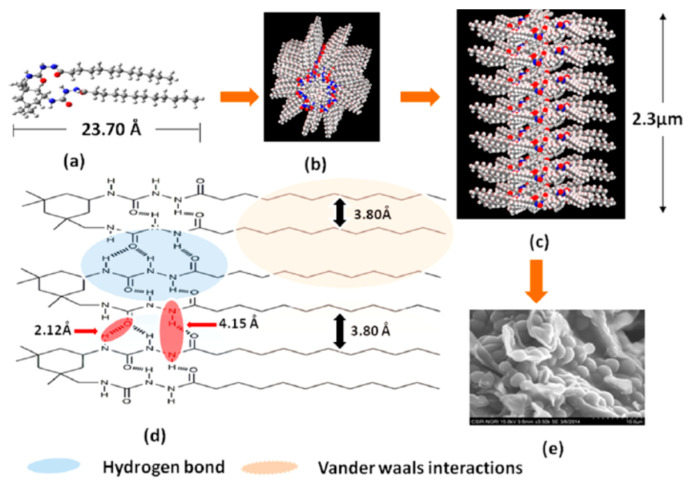
Schematic representation of the *C*_12_ gelator showing the (**a**) molecular length, (**b**) model of the molecular aggregate, (**c**) model of the probable mode of packing within the gel, (**d**) cross section and (**e**) xerogel SEM image. Reprinted with permission from ACS [45].

The combined experimental and computational study on these gels revealed that the balance of hydrogen bonding between the amido groups in acyl-semicarbazide moieties and van der Waals forces between long alkyl chains was the determining factor in the gelation process and influenced the morphology of the aggregates in each case. Both intra and intermolecular hydrogen bonding stabilized the structures and were predominant with shorter chain lengths (*C*_8_–*C*_14_) but van der Waals interactions increased in derivatives with longer chains (*C*_14_–*C*_18_). Figure 15 shows, as an example, the two non-covalent forces and their influence on the mode of aggregation of the *C*_12_-derivative.

## 3. Cyclobutane Derivatives

In this section, comments about organogelators based on cyclobutane-containing bisamides, which will be compared with the cyclohexane partners, along with alkyl amino acid derivatives and peptidomimetics are provided.

### 3.1. Bisamides

**Figure 16:**
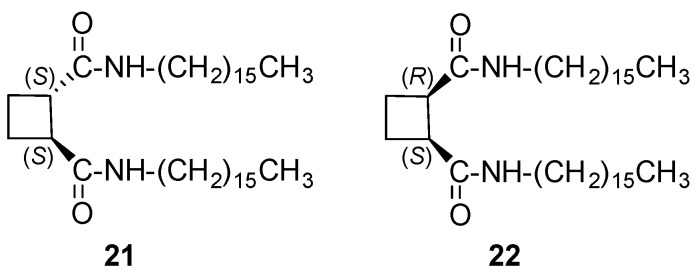
Cyclobutane-containing bisamide organogelators based on cyclobutane-1,2-dicarboxylic acid.

Chiral and achiral meso derivatives **21** and **22** (Figure 16) were prepared from commercially available (1*R*,2*R*)- and (1*R*,2*S*)-cyclobutane-1,2-dicarboxylic acid, respectively and their behavior as organogelators was compared with that of cyclohexane derivatives **11** and **12** (see Section 2.1) [33].

**Figure 17:**
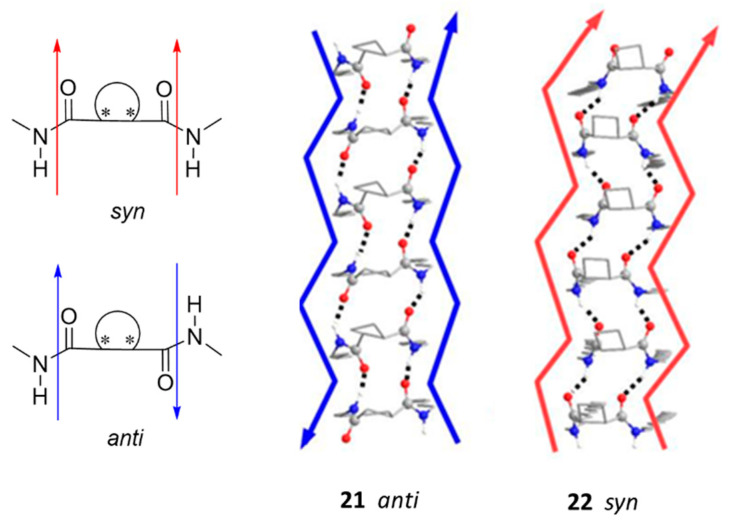
**Left**: *syn* and *anti*-disposition of amido groups. Asterisks represent the stereogenic centers. **Right**: predicted structures of the octameric aggregates of compounds **21** and **22**; nonpolar hydrogen atoms have been omitted for clarity. Adapted with permission from Wiley [33].

Compounds **21** and **22** were worse organogelators than cyclohexane-based derivatives **11** and **12** but they were selective for toluene with *cgc* around 50 mg mL^−1^. CD confirmed that both aggregates were chiral despite monomer **22** was not; CD spectra of the xerogels showed a bisignate positive Cotton effect for the xerogel of **21** and a negative one for that of **22**, from toluene. While *cis/trans* stereochemistry had no influence on their gelation ability of apolar solvents, the morphology of their xerogels was different as shown by SEM: *trans* **21** formed disordered fibers of around 40 µm and *cis* **22** was self-organized into spheres of different sizes between 10 and 20 µm. Computational calculations suggested that the driving forces to form ordered aggregates were promoted by the cooperative effect of both hydrogen bonding and van der Waals interactions, which had similar contributions. Calculations also suggested that trans **21** displays an *anti*-disposition of the amido groups, whereas cis **22** showed a syn one. Both monomers self-assemble to form ribbon-like aggregates with a zigzag arrangement in both cases, but with a certain helicity in the case of **22** (Figure 17).

### 3.2. Amino Acid Derivatives with Alkyl Tails

**Figure 18:**
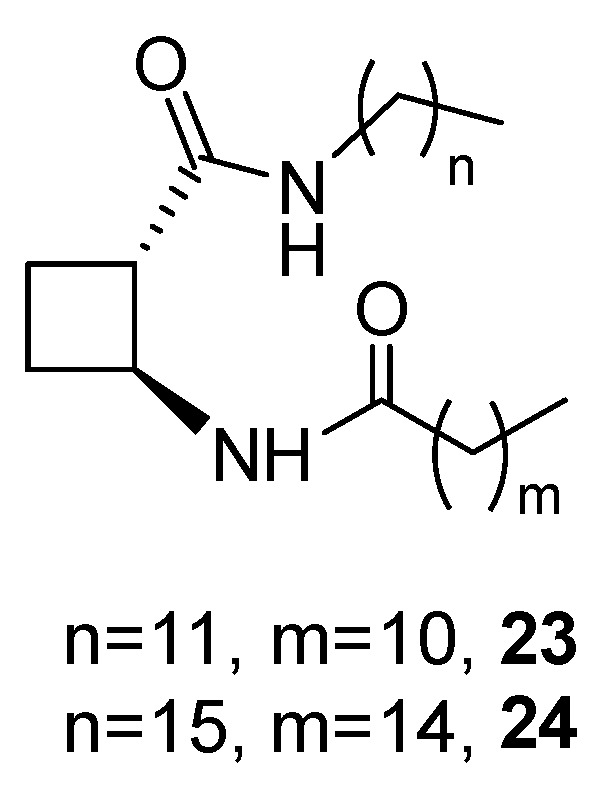
Cyclobutane-containing amino acid derivatives with alkyl tails.

Derivatives **23** and **24** bearing *C*_12_ and *C*_14_ tails, respectively, (Figure 18) were synthesized from previously reported (1*S*,2*S*)-2-aminocyclobutanecarboxylic acid (CBAA) [46]. In contrast with bisamides **21** and **22**, which were insoluble in alcohols [33], these compounds were good gelators for methanol, ethanol and isopropanol (*cgc* values between 8–47 mg mL^−1^) and for biocompatible ethanol-water mixtures (up to 30% water) with *cgc* = 10–16 mg mL^−1^ [47].

### 3.3. Peptidomimetics

**Figure 19:**
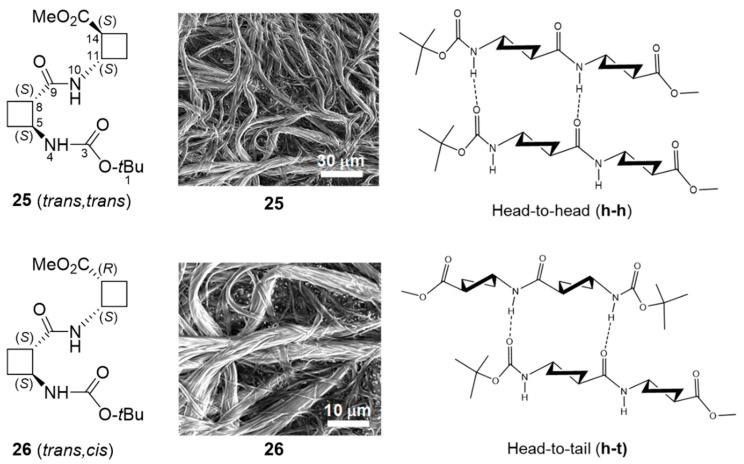
Chemical structures of dimeric CBAA derivatives **25** and **26** (**left**), SEM images of samples of **25** and **26** as xerogels from toluene (**center**) and the possible arrangement of two molecules of **25** interacting through two intermolecular hydrogen bonds (**right**). Adapted with permission from Wiley [49].

The ability of dimers derived from cyclobutane-containing β-amino acids, CBAAs, either combining *trans*, *trans*- and *trans*, *cis*-diastereomers, respectively, was investigated. Peptidomimetics **25** and **26** (Figure 19) folded in chloroform solution giving well defined conformers as the result of intramolecular and inter-residue hydrogen-bonding [48]. Nevertheless, they manifested some tendency to aggregate to give fibrils from methanol solutions as verified by TEM and Atomic Force Microscopy (AFM). Moreover, gels of **25** and **26** were obtained from 40 mM solutions in toluene and in 1:1 mixtures of dichloromethane/pentane or ethyl acetate/pentane. The influence of the *cis/trans* stereochemistry and the statement of the non-covalent interactions leading to the formation of helical aggregates was explored by the combination of experimental techniques and computational calculations [49].

^1^H NMR in toluene-*d*_8_ allowed to determine *T*_gel_ for both organogellators that was around 270 K at 15 mM; the spectroscopic study indicated a conformational change of the molecules in the aggregate with respect to the preferred conformation of single molecules as well as a different packing for the gels from the two peptides. Computational calculations suggested that the assemblies are the result of hydrogen-bond formation between the two NH groups of one molecule and the carbonyl groups at positions 3 and 9 of the other one (see Figure 19 for the numeration). These hydrogen-bonds propagate along the aggregate axis to induce a helical mode of aggregation, which was supported by the CD spectral signature of the xerogels that displayed a negative Cotton effect at 213–214 nm and a positive one at 233–234 nm. By other side, the orientation of two consecutive molecules in a single hydrogen bonded chain was head-to-head for dipeptide **25** and head-to-tail for **26**. These interactions involve a conformational change of the molecules, leading to a less-twisted form than those in solution, which is also in agreement with CD: the bands observed for **25** and **26** in solution were much more intense than those for the xerogels. The single chains interact with one another in an antiparallel way to afford bundles as shown by SEM, the significant geometry parameters of which fitted well to the main peaks observed in wide-angle X-ray diffraction spectra of the aggregates in the solid state.

**Figure 20:**
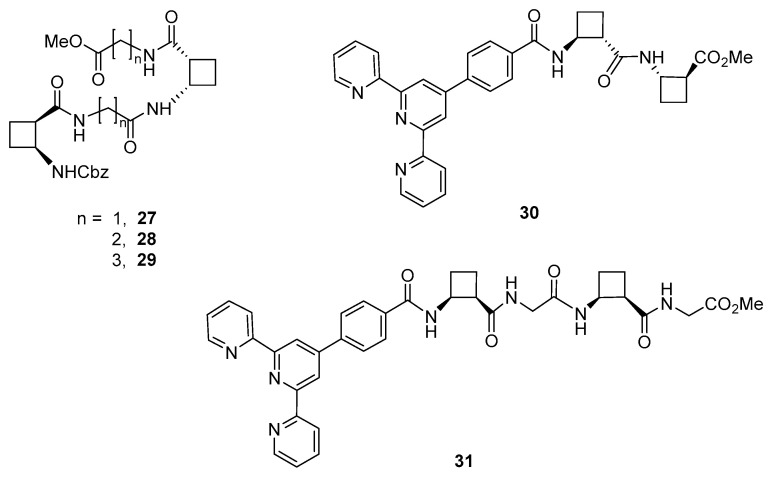
Chemical structures of hybrid peptides **27**–**29** and terpyridine-conjugated peptides **30** and **31**.

To investigate the role of molecular flexibility in the aggregation capabilities of CBBA-containing organogelators, hybrid peptides **27**–**29** (Figure 20) were synthesized and studied. They consist of *cis* (1*R*,2*S*)-CBBA and glycine, β-alanine, or γ-aminobutyric acid joined in alternation [50]. Peptides constituted only by *cis*-CBBA had shown to be moderated gelators for some few solvents [51]. In contrast, **27**–**29** bearing flexible linear spacers, afforded nice and stable gels in a broad range of solvents including alcohols, acetone, acetonitrile, tetrahydrofuran and 1,4-dioxane. The best *cgc* values were achieved in toluene (3–6 mg mL^−1^) and ethyl acetate (8–10 mg mL^−1^). Peptide **27** was the only gelator in chloroform where **28** and **29** were soluble. Gels from the three compounds were thermoreversible in toluene, acetonitrile, dichloromethane and, in the case of **27**, also in chloroform. On the contrary, gels in alcohols and ethers were not thermoreversible. SEM and CD pointed out that peptide **27**, which contains the shortest *C*_2_ linear residue, presented the most defined fibril network and afforded nanoscale helical aggregates in toluene. Tetrapeptide **29**, bearing *C*_4_ linear residues, also showed bundles of fibers whereas a homogeneous spherulitic network was observed for **28**, with a *C*_3_-spacer between cyclobutane residues. Computational calculations for **27** led to model the self-assembly of the molecules and suggested a head-to-head arrangement to give helical structures corresponding to hydrogen-bonded single chains.

To account for the cooperativity of an aromatic moiety with hydrogen-bonding interactions on the aggregation of cyclobutane-containing peptides, 4′-phenylterpyri- dine-conjugate derivatives **30** and **31** (Figure 20) were prepared and investigated [52]. These two-component systems were insoluble in very apolar solvents, such as pentane and in water but they formed gels in 1,4-dioxane, toluene, ethyl acetate, tetrahydrofuran, alcohols, acetone and acetonitrile. Compound **30** also gelated chloroform and dichloromethane whereas **31** was soluble. Macroscopically, the gels formed in alcohols were opaque, while the gels formed in other solvents were translucid. All gels were stable at room temperature, at least, for one month and they were thermoreversible.

For conjugate **30**, the lowest *cgc* values found were 49, 58 and 59 mg mL^−1^, corresponding to toluene, acetone and methanol, respectively. It is interesting that, although some *cgc* values were higher than those for the same solvents with dipeptide **25** as organogelator (Figure 17), **30** can gelate a broader range of solvents including apolar dichloromethane and protic polar solvents such as methanol and *tert*-amyl alcohol. In turn, **31** was insoluble in acetonitrile but soluble in chloroform and dichloromethane, but it could gelate other eight solvents tested. The best *cgc* values found were 43 and 48 mg mL^−1^, corresponding to isopropanol and methanol, respectively, which were also gelated by tetrapeptide **27** but with higher *cgc* values. In conclusion, the presence of the 4′-phenylterpyridine moiety allowed tuning the gelling properties and also influenced the supramolecular self-assembling mode to produce chiral aggregates with respect to parent compounds **25** and **27**.

**Figure 21:**
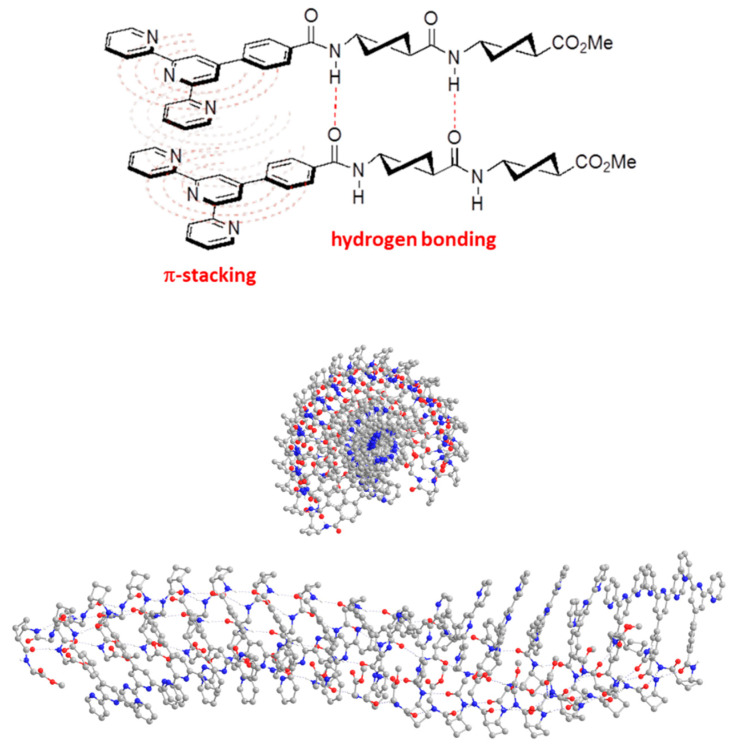
**Top**: Schematic representation of head-to-head arrangement of a dimer of conjugate **30**. π–π and H-bonding interactions are highlighted in red. **Bottom**: Top and side view for the predicted structure of a dodecameric aggregate of **31**. Non-polar H atoms were omitted for clarity. Adapted with permission of Elsevier [52].

Indeed, SEM experiments revealed that the morphology of the aggregates was solvent-dependent and less defined than for **25** and **27**. In toluene, they formed helical aggregates as verified by CD spectroscopy and supported by computational calculations (Figure 21). In summary, it was concluded that π–π interactions between the terpyridine substructures and hydrogen bonding between the amide groups of the peptides in consecutive molecules have a cooperative effect in the supramolecular arrangement to self-assemble into the corresponding aggregates.

**Figure 22:**
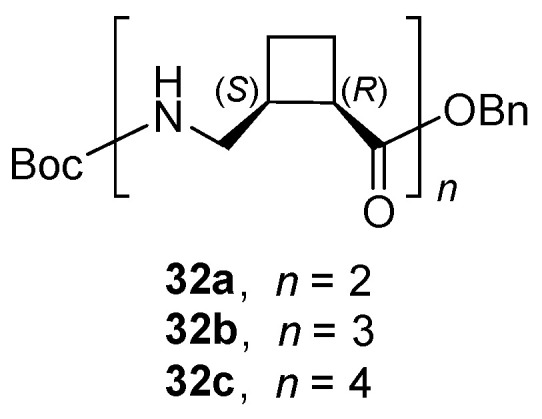
Chemical structure of short oligomers containing (1*R*,2*S*)-2-(aminomethyl) cyclobuta- ne-1-carboxylic acid as its repetitive unit.

Furthermore, the ability as organogelators of peptidomimetics made up with less rigid cyclobutane-containig γ-amino acids has also been examined [53]. Short oligomers **32a**–**c** were synthesized from *cis* (1*R*,2*S*)-2-(aminomethyl)cyclobutane-1-carboxylic acid [54] (Figure 22).

Among other conformations, these oligomers adopt ribbonlike folding in solution, prompted by the formation of intramolecular seven-membered hydrogen bonds. Dimer **32a** was not able to form gels. In contrast, **32b,c** formed thermoreversible fibrillar gels in concentrated solutions of different solvents. Peptide **32b** was better organogelator than tetramer **32c**, affording gels in toluene, THF, isopropanol, ethyl acetate and acetonitrile.

## 4. Cholesterol-Based Organogelators

Cholesterol displays unique features due to the chiral carbon-tetracyclic structure as a hydrophobic and rigid core favoring the molecular stacking through van der Waals interactions and with the possibility to bear functional groups and anchor alkyl chains or other structural units such as donor or acceptor systems. These characteristics made the cholesterol-based organogelators appropriate to produce gels with tunable properties and stimuli responsive materials [55,56].

**Figure 23:**
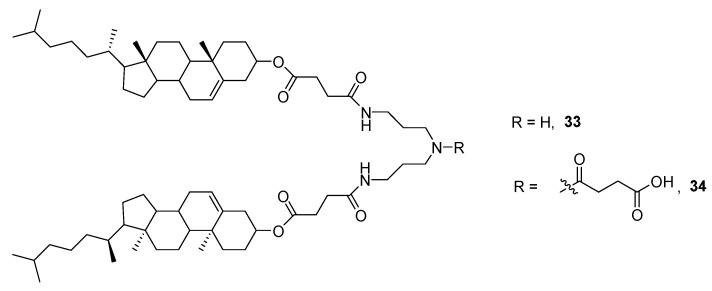
Examples of cholesterol derivatives examined as potential gelators.

Efforts have been devoted to correlate and rationalize molecular structure and gelation behaviors of these organogelators. For instance, compound **34** holding a carboxyl group (Figure 23) was able to gelate benzene and xylenes at *cgc* ≤ 20 mg mL^−1^ but neither **33** nor a product related to **34**, but with only one cholesteryl moiety, was not [57].

**Figure 24:**
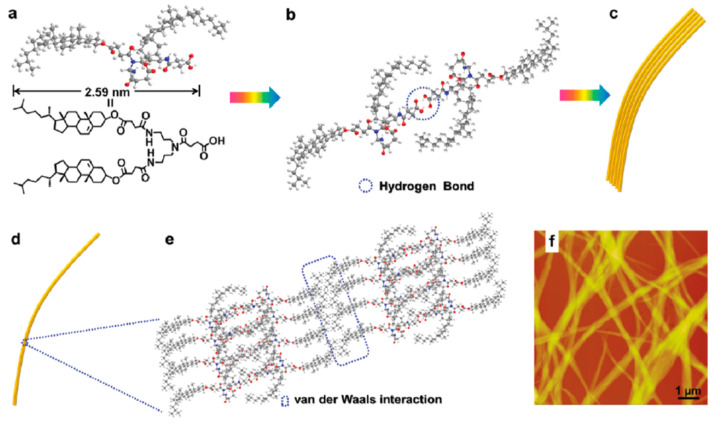
(**a**) Molecular structure of compound **34**; (**b**) molecular structure of the dimer of **34**; (**c**) fibers; (**d**) single nanofiber; (**e**) probable molecular packing model of **34** in a fiber and (**f**) the AFM image of the xerogel obtained from **34** in toluene (*cgc* = 10 mL^−1^). Reprinted with permission from CNRS and RSC [57].

Based on the molecular structure of **34**, the morphological observations by TEM and AFM, the Fourier transform infrared (FT-IR) spectroscopy and X-ray-diffraction studies, authors suggested a probable molecular packing model of **34** in the aggregates (Figure 24).

According to this model, the dimer would be formed through intermolecular hydrogen bonding between the carboxyl groups. In turn, the single nanofibers were produced via van der Waals interactions between the cholesteryl units. Thus, the fibers were formed by combining bundles of single nanofibers, which intertwined with one another to form a tridimensional network and, finally, leading to the gel.

**Figure 25:**
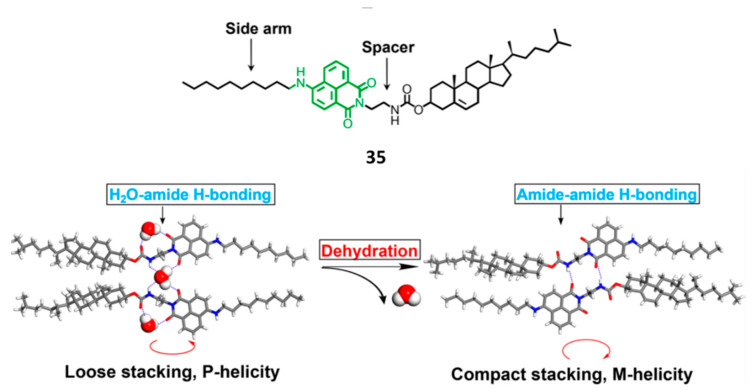
Chemical structure of compound **35** and proposed mechanism of dehydration-induced chirality inversion. Adapted with permission from ACS [58].

On the other hand, due to its eight stereocenteres, the cholesteryl moiety has been used in several organogelators to induce specific chirality in environment-adapted self-assembled systems [58,59]. For an instance, Figure 25 shows the structure of compound **35** consisting of a cholesterol substructure linked to a side-arm-bearing maleimide moiety through a spacer containing a carbamate group as potential hydrogen bonding site [58,60]. This compound and other related derivatives with *C*_3_-*C*_13_ side arms and *C*_1_-*C*_2_ short spacers, presented a case of induced chirality inversion by means of a water-binding-mediated gelation/crystallization [61].

**Figure 26:**
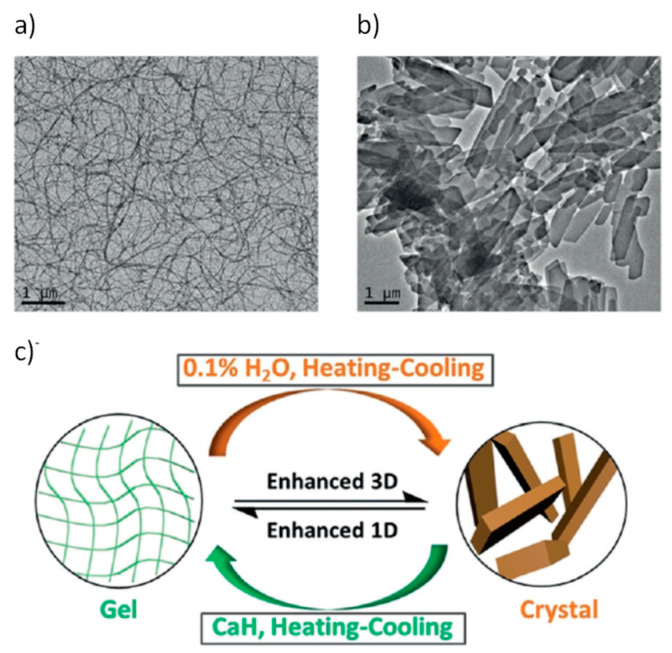
(**a**) TEM image of **35** self-assembly in decane without water; (**b**) TEM image of **35** micro-crystals induced by a trace amount of water; (**c**) representation of the phase transformation between gel and crystal states. Reprinted with permission from Wiley [61].

The first experiments showed that **35** formed vesicles in aqueous media and that mirror CD spectra of **35**-based assembly appeared upon gradual heating, thus implying chirality inversion. X-ray crystallography analysis revealed the participation of water molecules in the vesicles through interaction with the NH protons of the carbamate and additional coordination to the maleimide carbonyl groups leading to the formation of hydrogen-bonded aggregates. Under heating, dehydration takes place producing compact stacking between the cholesteryl moiety and the side arm of consecutive molecules further to the intermolecular hydrogen bonds between the carbamate groups. This change induced helicity inversion, which was verified, in addition to the CD spectra, by TEM images of the self-assemblies in THF-hexane and THF-water mixtures. Indeed, **35** gelated different organic solvents such as tetrahydrofuran or pentane, in absence of traces of water, affording infinite aggregated nanofibers, which were highly intertwined in the gels. Nevertheless, crystals were formed in the presence of even less than 0.1 vol% water (Figure 26).

**Figure 27:**
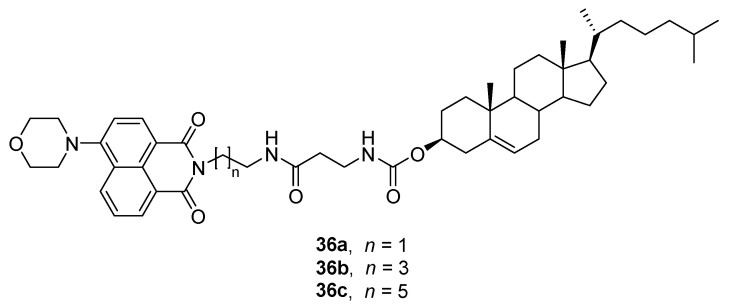
Chemical structure of cholesterol-based organogelators **36a–c**.

Otherwise, tunable gel formation was achieved by sonication or thermal stimulus using a family of cholesterol based fluorescent compounds **36a–c** containing naphthalimide connected by two acylamines and alkyl-chain spacers (Figure 27) [62].

These compounds were able to gelate alcohols, acetonitrile, ethyl acetate or *p*-xylene with *cgc* ≤ 25 mg mL^−1^. **36a** and **36c** were better gelators than **36b** pointing out the influence of the spacer length. The morphology and nature of the self-assemblies (fibers, vesicles) changed from the thermodynamic gel, obtained upon heating and subsequent cooling and the sonication-gel, produced by treatment of the solution with ultrasounds at room temperature. This means that the balance between the multiple driving forces for self-assembly, that is, hydrogen bonding, hydrophobic interactions and π–π interactions is altered by the ultrasound irradiation that provides heat and pressure on the nanosecond scale to selectively tidy the competing non-covalent interactions.

**Figure 28:**
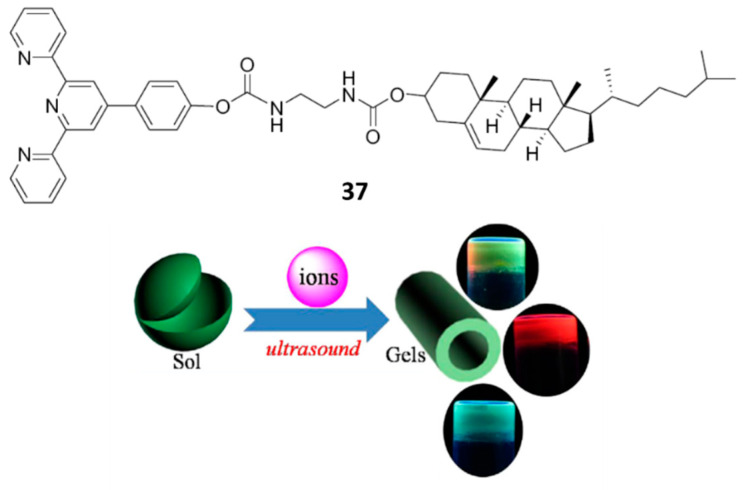
Structure of organogelator **37** and summarized formation of hybrid materials in the presence of ions and ultrasound activation. Adapted with permission from Elsevier [63].

Hybrid materials consisting of gels from organogelator **37** (Figure 28) and inorganic species were produced under ultrasounds-controlled sol-gel transition. The resultant gels showed emission color tunable properties based on ion identification [63]. The structure of **37** consists in a cholesterol moiety and an *O*-substituted terpyridine motif linked through two carbamate groups connected to a *C*_2_-flexible spacer.

Under heating-cooling, **37** was soluble in alcohols, ethyl acetate, benzene, or cyclohexane but it formed gels in these solvents under ultrasound stimuli even at room temperature. In ethanol, the lowest *cgc* (5 mg mL^−1^) and the highest gel-sol transition temperature (*T*_g_, 53 °C) were displayed. In addition, gel in ethanol exhibited self-healing properties, that is, when two separated pieces of cut gel were contacted within half an hour, they adhered with each other to one block again [64]. The driving force for the formation of organogels was attributed to π–π stacking and hydrogen bonding, enhanced by ultrasounds, as well as hydrophobic interactions.

**Figure 29:**
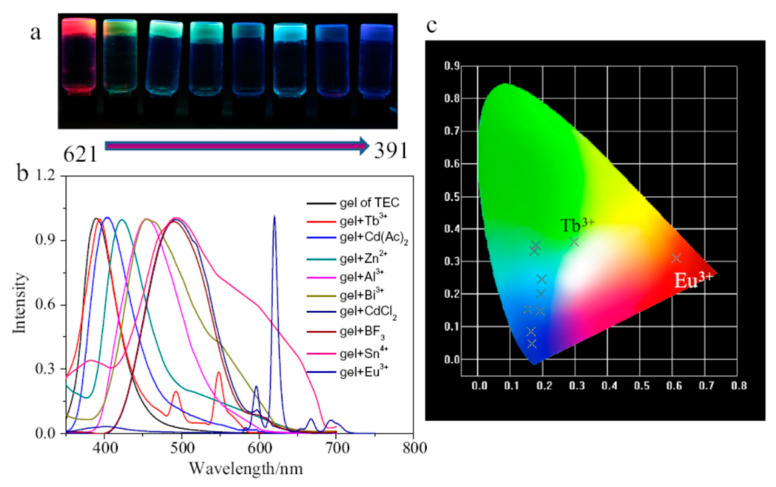
(**a**) The photos of **37**/ion hybrid gels in dark (irradiated at 365 nm), added ions from left to right: Eu(NO_3_)_3_, SnCl_4_, BF_3_.OMe_2_, CdCl_2_, Al(NO)_3_, Tb(NO)_3_, Zn(NO)_3_, Cd(AcO)_2_; (**b**) normalized fluorescence spectra of these hybrid gels; (**c**) CIE chromaticity diagram of the hybrid gels. Reprinted with permission from Elsevier [63].

Upon the addition of different metal salts (equimolecular amounts) gels could be also formed triggered by sonication and displaying tunable emission colors. The terpyridine moiety was useful to coordinate metal ions. Figure 29 shows the emission spectra of **37** hybrid gels in the presence of different metal ions and the CIE (National Commission on Illumination) chromaticity coordinates. As expected, ionic valences influenced on the fluorescent properties of the hybrid gels. Their morphology also changed with respect to that of the organogel due to the introduction of coordination interactions and the changes in hydrophilic and hydrophobic properties.

**Figure 30:**
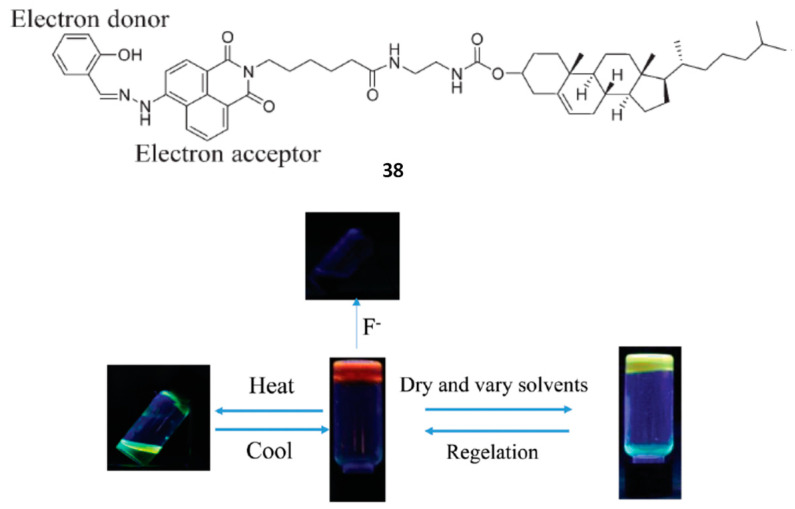
Chemical structure of organogelator **38** and photos of the fluorescent changes in its gels in the dark (irradiated at 365 nm) triggered by multiple stimuli. Adapted with permission from RSC [65].

Multicolor emissions in a monocomponent gel system were accomplished by varying the solvent and the temperature by using the cholesterol-based organogelator **38**. This molecule contains salicylaldehyde and naphthalimide units as a donor-acceptor pair, which is able to form charge transfer complexes (Figure 30) [65].

Under the classical heating-cooling protocol, gels were formed only in ethanol and benzene but **38** was able to gel short-chain alcohols, dichloromethane and acetone triggered by ultrasound irradiation at room temperature, with *cgc* values around 5 mg mL^−1^. Gels obtained under both activation types displayed transition in emission color with changing solvent and the morphology of their self-assemblies were also different as shown by SEM. All gels in different organic solvents were chiral. They were responsive to fluoride anion inducing changes in phase, color and fluorescence due to hydrogen bonding between fluoride anions and NH and OH groups, which hindered the charge transfer process of naphtalimide.

**Figure 31:**
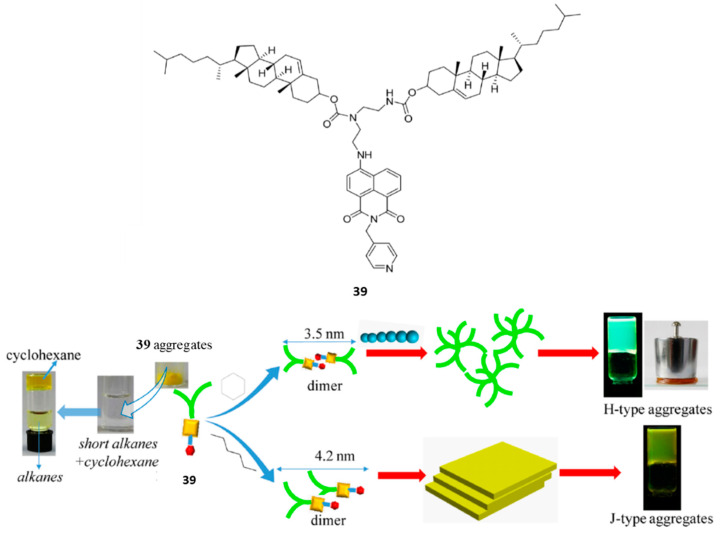
Top: chemical structure of gelator **39**. Bottom: proposed gelation processes and aggregation difference of **39** in cyclohexane and hexane and illustration of **39** xerogel for cyclohexane uptake from solvent mixtures. Adapted with permission from ACS [66].

In another work from the same laboratory, with the aim of achieving visual and selective discrimination of organic solvents with similar polarity, a self-healing and multistimuli responsive cholesterol-based supergelator, **39**, containing a naphtalimide unit as the electron acceptor and a pyridine unit as the electron donor (Figure 31) was reported [66].

Compound **39** formed stable, transparent and green gels in cyclohexane with *cgc* = 0.18 wt %, which fell into the category of supergelators (<0.2 wt %). The instant gel-formation was prompted both by heating-cooling, ultrasound, shaking-rest and staying at room temperature. In contrast, only heating-cooling followed by sonication prompted the formation of an opaque and yellow gel in hexane. No gelation occurred in other tested organic solvents. The gel in cyclohexane presented a fluorescence maximum emission peak at 524 nm and normalized intensity value of 6686, while the peak of the gel in hexane was red shifted to 54 nm, with intensity 2238. Therefore, cyclohexane and hexane can be discriminated visually by different emission colors in the gels this suggesting different aggregation modes of the fluorophore. For the gel in hexane, UV-vis experiments indicated J aggregates of the fluorophore. Contrarily, for **39** in cyclohexane, the 9 nm blue-shift from the solution to the gel reflected the enhanced π–π stacking interaction with the H-type aggregation mode. In an H-aggregate, molecules stake predominantly face-to-face while J-aggregates form when molecules primarily stack in a head-to-tail arrangement [67,68]. The morphology of the aggregates in each solvent was also different as revealed by SEM and TEM. While the xerogels from cyclohexane showed a flower structure, which was composed of cross-linked nanospheres, multilayer sheet structures were observed for xerogels from hexane (Figure 28). Moreover, X-ray diffraction patterns revealed the formation of dimers suggesting a denser structure for the dimer in the xerogel from cyclohexane and a lamellar ordered structure from hexane.

Since the gel formation in a particular solvent can be considered as a balance between dissolution and self-assembly of a gelator, HSPs were calculated and analyzed. Results suggested that the dispersion interactions are the main factor for the selective gelation of **39** toward short-chain alkanes and that hydrogen-bonding force might be the major factor contributing to the specific and strong interaction between **39** and cyclohexane.

The gel in cyclohexane displayed self-healing properties and, in addition, it was sufficiently strong that it could be molded into self-sustaining and self-supporting geometrical shapes. These properties confer on it with interesting possible applications as, for instance, to repair biological tissues. Unlikely, the gel of **39** in hexane could not be molded to any self-supported block.

Despite there are several examples in the literature about the oil/water separation in two-phase solvent mixtures through the gel formation approach [5], gelator **39** afforded the first example reported to separate a specific organic solvent from single-phase solvent mixtures. Indeed, cyclohexane could be separated within 10 min from complex mixtures including short-chain alkanes by using the **39** xerogel.

Finally, the gel of **39** in cyclohexane displayed responsive properties to chemical stimuli because it was proved to be a high-performance sensor toward gases of HCl and NH_3_.

## 5. Summary and Conclusions

Simple carbocycle-based organogelators, mainly in their chiral version, are useful to produce gels with tunable properties making them suitable for multiple purposes. The balance or the cooperation between the non-covalent forces leading to aggregates can be monitored by the introduction of hydrogen-bonding functional groups, alkyl-chains and other structural units such as aromatic moieties. Both chirality, conformational bias and molecular rigidity are parameters controlling molecular stacking and, consequently, the specific features of self-assemblies. The energy source for gelation, i.e., thermal or ultrasound, sometimes plays essential roles being responsible for chirality induction on the aggregates, for their morphology, or for the production of self-healing gels. Cholesterol-based organogelators are specially interesting and useful for multiple applications. The possibility to link the cholesteryl moiety to donor/acceptor systems through flexible segments containing functional groups allows the design of sophisticated monocomponent gel systems and hybrid gels. These characteristics confer on these organogelators with a great versatility to produce gels with modulated abilities or stimuli-responsive materials.

## Data Availability

Not applicable.
